# Differential contribution of THIK-1 K^+^ channels and P2X7 receptors to ATP-mediated neuroinflammation by human microglia

**DOI:** 10.1186/s12974-024-03042-6

**Published:** 2024-02-26

**Authors:** Ali Rifat, Bernardino Ossola, Roland W. Bürli, Lee A. Dawson, Nicola L. Brice, Anna Rowland, Marina Lizio, Xiao Xu, Keith Page, Pawel Fidzinski, Julia Onken, Martin Holtkamp, Frank L. Heppner, Jörg R. P. Geiger, Christian Madry

**Affiliations:** 1 Institute of Neurophysiology, Charité – Universitätsmedizin Berlin, corporate member of Freie Universität Berlin and Humboldt-Universität zu Berlin, Charitéplatz 1, 10117 Berlin, Germany; 2 Berlin Institute of Health at Charité – Universitätsmedizin Berlin, Charitéplatz 1, 10117 Berlin, Germany; 3 Cerevance Ltd, 418 Cambridge Science Park, Milton Road, Cambridge, CB4 0PZ UK; 4 Department of Neurology, Epilepsy-Center Berlin-Brandenburg, Charité - Universitätsmedizin Berlin, corporate member of Freie Universität Berlin and Humboldt-Universität zu Berlin, Charitéplatz 1, 10117 Berlin, Germany; 5 Neurocure Cluster of Excellence, Neuroscience Clinical Research Center, Charité – Universitätsmedizin Berlin, corporate member of Freie Universität Berlin and Humboldt-Universität zu Berlin, Charitéplatz 1, 10117 Berlin, Germany; 6 Department of Neurosurgery, Charité – Universitätsmedizin Berlin, corporate member of Freie Universität Berlin and Humboldt-Universität zu Berlin, Charitéplatz 1, 10117 Berlin, Germany; 7 Department of Neuropathology, Charité – Universitätsmedizin Berlin, corporate member of Freie Universität Berlin and Humboldt-Universität zu Berlin, Charitéplatz 1, 10117 Berlin, Germany; 8 German Center for Neurodegenerative Diseases (DZNE) Berlin, 10117 Berlin, Germany

**Keywords:** Ion channels, Microglia, Neuroinflammation, Human brain, Neocortex, Purinergic signalling, Pharmacology

## Abstract

Neuroinflammation is highly influenced by microglia, particularly through activation of the NLRP3 inflammasome and subsequent release of IL-1β. Extracellular ATP is a strong activator of NLRP3 by inducing K^+^ efflux as a key signaling event, suggesting that K^+^-permeable ion channels could have high therapeutic potential. In microglia, these include ATP-gated THIK-1 K^+^ channels and P2X7 receptors, but their interactions and potential therapeutic role in the human brain are unknown. Using a novel specific inhibitor of THIK-1 in combination with patch-clamp electrophysiology in slices of human neocortex, we found that THIK-1 generated the main tonic K^+^ conductance in microglia that sets the resting membrane potential. Extracellular ATP stimulated K^+^ efflux in a concentration-dependent manner only via P2X7 and metabotropic potentiation of THIK-1. We further demonstrated that activation of P2X7 was mandatory for ATP-evoked IL-1β release, which was strongly suppressed by blocking THIK-1. Surprisingly, THIK-1 contributed only marginally to the total K^+^ conductance in the presence of ATP, which was dominated by P2X7. This suggests a previously unknown, K^+^-independent mechanism of THIK-1 for NLRP3 activation. Nuclear sequencing revealed almost selective expression of THIK-1 in human brain microglia, while P2X7 had a much broader expression. Thus, inhibition of THIK-1 could be an effective and, in contrast to P2X7, microglia-specific therapeutic strategy to contain neuroinflammation.

**Graphical Abstract:**

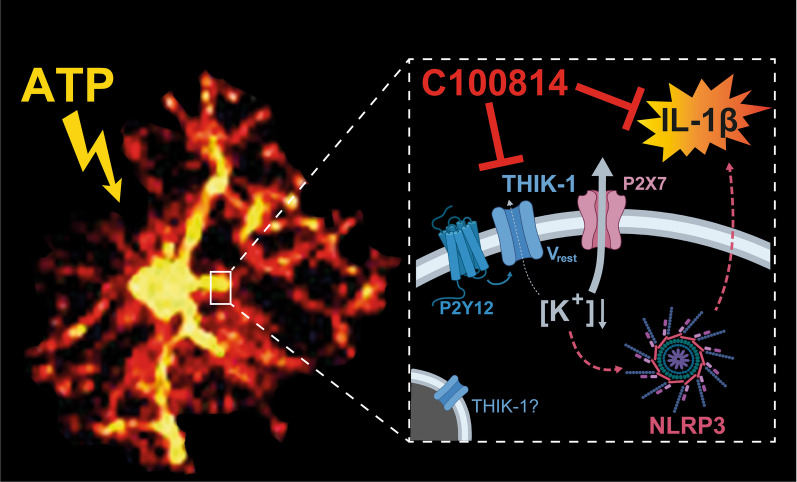

**Supplementary Information:**

The online version contains supplementary material available at 10.1186/s12974-024-03042-6.

## Background

Neuroinflammation, a condition affecting millions of people worldwide, is a major contributor to and risk factor for neurological diseases, including traumatic, ischemic, infectious, psychiatric and neurodegenerative diseases [1, 2]. However, currently approved drugs by the FDA or EMA to suppress neuroinflammatory processes are still very limited, targeting only a small spectrum of these diseases and primarily via indirect mechanisms [3, 4]. Research and development of therapeutic strategies aimed at containing neuroinflammation are therefore of paramount clinical importance. A promising novel approach focuses on manipulating microglia, the brain’s innate immune cells, which are involved in virtually all neurological diseases and thus have a significant impact on inflammatory processes [5–7].

Microglia protect the brain from injury and invading pathogens by triggering an inflammatory response aimed at containing damage and restoring homeostasis [8]. However, depending on the severity and duration of the pathological condition, microglia may also adopt proinflammatory states driving disease progression [9, 10]. Their pathological potential is significantly determined by the NOD-like receptor 3 (NLRP3) inflammasome, which is implicated in many neurodegenerative diseases, such as Alzheimer’s and Parkinson’s disease or amyotrophic lateral sclerosis (ALS) [11]. NLRP3 is a cytosolic multiprotein complex that assembles into caspase-1-activating platforms, which in turn catalyse the production of the proinflammatory cytokines IL-1β and IL-18 [12]. As a key signalling event, NLRP3 activation requires a sharp drop in intracellular [K^+^] to which many pathological stimuli mechanistically converge [13, 14]. Of these, extracellular ATP, the level of which rises significantly in brain pathology, is one of the most potent endogenous danger-signalling molecules triggering microglial activation [15, 16].

In immune cells, NLRP3 activation is closely tied to the activation of low-affinity ATP-gated P2X7 receptors, nonselective cation channels that generate K^+^ efflux along with Na^+^ and Ca^2+^ influx [17, 18]. In addition to P2X7, the two-pore domain K^+^ channel THIK-1 was recently identified to regulate the release of IL-1β in response to purinergic stimulation from rodent microglia [19, 20]. Apart from its tonic activity, THIK-1 can also be potentiated by a rise in ambient ATP level due to metabotropic coupling to high-affinity P2Y12 receptors [19]. Although it was hypothesized that THIK-1 affects IL-1β release by lowering intracellular K^+^ for NLRP3 activation [19, 20], its role in regulating K^+^ gradients in this context remains unknown, as do its interactions with P2X7 or other ATP-gated ion channels. The combination of a nonselective cation channel (P2X7) with a tonically active potassium channel (THIK-1) may indeed be advantageous due to the increased driving force for K^+^ efflux via THIK-1 at more depolarizing membrane potentials that results from P2X7 activation. Therefore, it is essential to investigate the role of THIK-1 in the context of all K^+^-permeable ion channels that are activated in microglia upon a rise of [ATP] which triggers NLRP3 activation.

Current knowledge of microglial physiology still relies heavily on experiments using rodent models and cell culture, whereas studies examining microglial function in human brain tissue are extremely sparse [21, 22], mainly due to limited access to surgically removed brain tissue and difficulties in experimental handling. This is particularly problematic as transcriptomic studies have revealed significant differences in the genetic profile between rodent and human microglia [23, 24]. Moreover, microglial properties are influenced by the type of preparation, because microglia lose their characteristic genetic signature when removed from the brain and transferred into culture [25]. Not surprisingly, this can lead to significant and species-dependent differences in physiological and pathophysiological functions [26, 27]. This is reflected, for example, in different functional properties and isoforms between rodent and human P2X7 receptors [28], for which there is surprisingly little direct electrophysiological evidence in microglia in rodent brain tissue [29, 30] and none in the human brain. Of note, the poor transferability of preclinical results from animal models to humans is a major hurdle in drug development and a possible contributing factor to the failure of many clinical trials [31, 32]. To assess the potential therapeutic value of THIK-1, it is therefore important to examine the relevant mechanisms in the human brain, which have not been addressed thus far.

Here, we characterised the electrical membrane properties and K^+^ permeable ion channels underlying ATP-induced NLRP3 activation in microglia in slices of acutely resected human neocortex by patch-clamp electrophysiology. By introducing C100814, a novel, specific THIK-1 inhibitor and a prerequisite in preparations where genetic manipulations (i.e., knock-out) are not feasible, we investigated the role of THIK-1 and its interactions with P2X7 in NLRP3-mediated IL-1β release by immunological assays and genetic profiling. Due to the potent suppression of IL-1β release by C100814 and selective expression of THIK-1 in human microglia compared to P2X7, our findings imply a preferential role of THIK-1 antagonists that may be employed therapeutically in containing neuroinflammation.

## Methods

### Compounds and reagents

Synthesis and chemical details of C100814 are described in Additional file 1. Drugs were obtained from Sigma-Aldrich or, for MCC950 and A740003, from Tocris. Application of drugs in electrophysiogical experiments was via bath perfusion or locally via pressure ejection from a puffing pipette located at the slice surface (in the case of repeated brief ATP applications).

### Human brain tissue

For all experiments, except NETSseq, neocortical tissue was obtained from temporal lobe resections of 9 patients diagnosed with drug-resistant epilepsy (5 male, 4 female, age range 17 to 47 years) using only tissue that was removed to access the disease focus.

For NETSseq experiments, human tissue samples were obtained from the Miami Brain Endowment Bank (University of Miami), the General section of the Douglas-Bell Canada Brain Bank (Douglas Bell Hospital Research Centre, Montreal, Quebec, Canada), the NIH NeuroBioBank at the University of Miami, Maryland Brain Bank and Mount Sinai/JJ Peters VA Medical Center NBTR, Tissue4Research, London Neurodegenerative Diseases Brain Bank at King’s College London (LNDG receives funding from the UK Medical Research Council and as part of the Brains for Dementia Research Program, jointly funded by Alzheimer’s Research UK and the Alzheimer’s Society), The Netherlands Brain Bank (NBB, Netherlands Institute for Neuroscience, Amsterdam) and the South West Dementia Brain Bank (SWDBB).

### Mice

Wild-type (WT, C57BL/6) and THIK-1 knockout (KO) mice [19] (generously generated and provided by MRC Harwell) of either sex aged 2–4 months were used. To visualize microglia for patch clamp experiments, WT and THIK-1 KO mice were cross-bred with *Cx3Cr1*^*GFP*^ reporter mice [33], which express enhanced green fluorescent protein (GFP) under the control of the endogenous Cx3Cr1 locus. Housing of mice was in individually ventilated cages under pathogen-free conditions.

### Human brain slice preparation

Temporal lobe tissue resected from the patients was transferred within 10–15 min from the operating theatre to the laboratory in sterile sucrose-based slicing solution containing (mM): 87 NaCl, 2.5 KCl, 3 MgCl_2_, 0.5 CaCl_2_, 10 glucose, 75 sucrose, 1.25 NaH_2_PO_4_, and 25 NaHCO_3_, pH 7.4, 310 mOsm/l, oxygenated with 95% O_2_/5% CO_2_ at < 4 °C. After removal of the pia mater, the tissue was cut into 300 μm thick slices in ice-cold slicing solution. This was followed by a 30 min recovery period at 34–36 °C, after which slices were stored in slicing solution at room temperature until experimental use.

### Mouse brain slice preparation

Mice were decapitated under isoflurane anaesthesia. Whole brains were rapidly removed from the skull and immediately immersed in ice-cold sucrose-based slicing solution bubbled with 95% O_2_/5% CO_2_ (as for human tissue). Acute horizontal slices (300 μm) of the neocortex were prepared according to the protocol of Bischofberger et al. [34] (in cooled slicing solution at < 4 °C. Slices were allowed to recover in warmed, 34–36 °C slicing solution for 30 min and then kept at room temperature until experimental use.

### External and intracellular solutions

Slices were superfused with bicarbonate-buffered extracellular solution at 34–36 °C and a perfusion rate of 3–5 ml/min, containing (mM) 125 NaCl, 2.5 KCl, 25 NaHCO_3_, 1.25 NaH_2_PO_4_, 2 CaCl_2_, 1 MgCl_2_, and 10 glucose, bubbled with 95% O_2_/5% CO_2_. Drugs were added to the external solution or locally applied by a puffing pipette inserted into the slice as stated in the text.

Microglia were whole-cell patch clamped with an intracellular solution containing (mM) 140 K-gluconate or 130 KCl, 4 NaCl, 1 CaCl_2_, 1 MgCl_2_, 10 HEPES, 10 EGTA, 4 MgATP, and 0.5 Na_2_GTP, pH adjusted to 7.2 with KOH. Final osmolarity was 290 ± 5 mOsm/l.

### Identification of microglia for patch-clamp electrophysiology

Microglia in slices from mice were identified by their genetically encoded GFP tag by epifluorescence using a high-power LED light source (Thorlabs) at 488 nm excitation. Microglia in human slices were identified by their characteristic somatic morphology using infrared video microscopy on an upright Olympus BX51 microscope (with a 60× water immersion objective, NA 0.9) equipped with enhanced differential interference contrast (WI-DICT) and a high-sensitivity CMOS camera (Hamamatsu ORCAFlash LT). Specifically, microglial somata in human brain slices appear as small, oblong and dim-contrast structures of 5–10 µm in diameter, sometimes containing a characteristic dark spot at one of their apical endings. To avoid accidentally targeting astrocytes, which are similar in soma size, acutely counterlabelling astrocytes with 0.5 µM sulforhodamine 101 for 20 min [35] facilitates detection of the less abundant, unlabelled microglia. Regular training and close monitoring of morphological criteria resulted in an overall success rate per patch approach of > 90%. To limit confounding factors caused by unavoidable tissue damage during tissue resection and slicing, only microglia located at depths > 60 μm from the slice surface and within a period of 4–5 h after resection were analysed.

### Electrophysiological recordings of microglia

Microglia were whole-cell patch-clamped in brain slices using borosilicate pipettes with a tip resistance of 4–5 MΩ, giving a series resistance of < 20 MΩ. Voltage- and current-clamp recordings were performed using an Axopatch 200B amplifier (Molecular Devices). Currents were filtered at 1 kHz (10 kHz for membrane test), digitized (10 kHz) and analysed off-line using pClamp10 software with the researcher blind to experimental condition. Electrode junction potentials were compensated.

Input (Rt) and series resistances (Rs) were analysed from voltage clamped cells held at − 30 mV by applying 10 mV hyperpolarizing voltage steps using Ohm’s law (R = V/I).

Current–voltage (I–V) relations were from current responses to 80 ms voltage steps, starting from a holding potential of − 60 mV to allow activation of voltage-gated K^+^ channels if expressed.

For determination of the individual K^+^ conductance carried by THIK-1 K^+^ channels and P2X7 receptors during ATP-evoked NLRP3 activation, microglia were voltage clamped at 0 mV, corresponding to the approximate membrane voltage of microglia when P2X7 receptor activation occurs. Individual ion channel components were pharmacologically isolated by sequential application of 5 µM C100814 (to block THIK-1) followed by additional application of 10 µM A740003 (to block P2X7). Brief current transients in response to 150 ms hyperpolarizing voltage steps (− 40 mV) were triggered (i) prior to drug application and when reaching steady state after blockade of (ii) THIK-1 and (iii) additionally P2X7. Using Ohm’s law, individual conductances were determined by subtracting (i) − (ii) for THIK-1 and (iii) − (ii) for P2X7. Assuming the total K^+^ conductance is the sum of the individual THIK-1 and P2X7 conductance, their relative proportions were calculated, considering a K^+^ component of the P2X7-mediated current of 45%. This is based on the measured P2X7 reversal potential of ~ 0 mV, considering that P2X7 is approximately equally permeable to Na^+^ and K^+^, including significant Ca^2+^ permeability [17, 18].

### ELISA measurements of cytokine release

As previously described, brain slices of 300 µm thickness were prepared in ice-cold HEPES-buffered medium (MEM, pH 7.4, 42360-032, Gibco) under sterile conditions [19]. To induce inflammasome activation and IL-1β release, slices were exposed to ATP concentrations as stated in the text to simulate acute brain injury. Slices were placed on a Millicell cell culture insert (30 mm diameter, 0.4 µm pore size, PICM0RG50, Merck Millipore) and transferred into 6-well plates containing 1 ml serum-free medium (DMEM, pH 7.4, 41965-039, GIBCO) with or without lipopolysaccharide (LPS) (1 µg/ml, Escherichia coli 055:B5, L2880, Sigma-Aldrich), and/or inhibitors in a cell culture incubator at 37 °C. Slices were incubated for 6 h in total, with ATP as an activating stimulus added for the last 3 h. The amount of IL-1β released into the medium was measured by high-sensitivity ELISA using a Mesoscale MSD V-PLEX Proinflammatory Panel 1 Human (K15049D) and Mouse Kit (K15048D). To compare data between brain slices for different conditions, data were normalised to the mean of the control or ATP condition. Analysis was performed with the researcher blind to experimental condition.

### Nuclear enriched transcript sort sequencing (NETSseq)

All procedures including sorting of microglia, nuclei isolation, gene expression analyses and fixation of tissue samples were performed as previously described [36, 37]. Briefly, fixed and quenched nuclei were pelleted at 1.000×*g* for 4 min at 4 °C before washing once with homogenisation medium and once with wash buffer (PBS, 0.05% Triton X-100, 50 ng/ml bovine serum albumin, 1 mM DTT, 10 U/μl Superase-In RNase Inhibitor). Nuclei were treated for 30 min with blocking buffer (wash buffer supplemented with 50 ng/ml BSA) before the application of primary and secondary antibodies. Finally, nuclei were labelled with DAPI at a concentration of 0.01 mg/ml. Analysis and sorting of nuclei was performed using a BD FACSAria Fusion flow cytometer (BD Biosciences, San Jose, CA, USA) equipped with 355 nm, 488 nm, 561 nm, and 640 nm lasers. RNA quality was assessed according to the protocol by Brice et al. [38].

### Statistics

Data are presented as mean ± SEM (standard error of the mean). Statistical analysis was performed using OriginPro 2023. For normally distributed data, two-tailed Student’s t tests were performed, and for nonnormally distributed data, Mann‒Whitney U or Wilcoxon signed-rank tests were performed. The normality of the data was assessed using the Anderson‒Darling and Shapiro‒Wilk tests. The F test was used to confirm the equality of variance, and heteroscedastic t tests were used for unequal variance. To determine the appropriate sample size for a standard experiment, several factors were considered. These include a control response of 100%, a typical response standard deviation of 25%, an effect size of 50–90% (e.g. potency of inhibition), a desired power of 80%, and a significance level of p < 0.05. Based on these criteria, it was determined that < 6 cells are required in both groups (http://www.biomath.info/power/ttest.htm). However, actual numbers may vary for different experiments depending on effect size and standard error. *P* values < 0.05 were considered significant.

## Results

### THIK-1 regulates the resting potential of microglia in the human brain

**Fig. 1:**
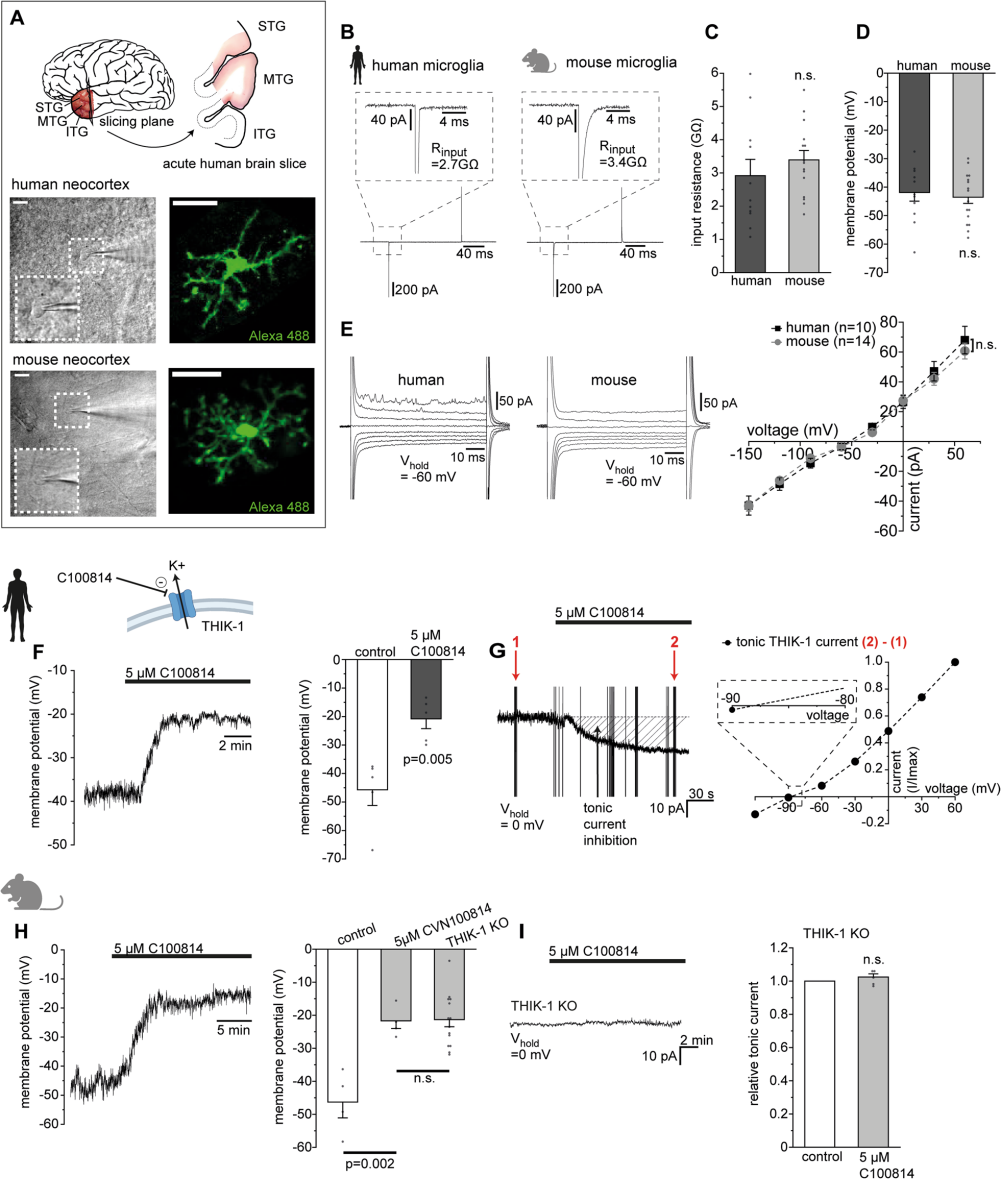
THIK-1 regulates the electrical membrane properties in microglia in the human brain. **A** Top: anatomical localisation of human temporal lobe from which acute brain slices were prepared. Bottom: specimen images of morphologically identified microglia patch clamped in human (top) and murine (bottom) brain slices (scale bar, 50 µm) and after filling them with Alexa-488 via the patch solution (scale bar, 20 µm). **B** Membrane currents with capacitive and leak components in response to 10 mV hyperpolarizing voltage steps of human und murine microglia, from which electrical membrane properties were determined. **C**, **D** Comparison of input resistance (**C**), and resting membrane potential (**D**) of human and murine microglia. **E** Specimen current profiles in response to voltage steps from − 150 to + 60 mV of a human and murine microglia (left) and respective plot of voltage dependence for all cells per condition (right). **F** Effect of THIK-1 inhibitor, C100814, on the resting membrane potential of human microglia, illustrating the time course of changes of an individual cell (left) and the resulting mean depolarization (right). **G** Specimen recording showing C100814-induced changes in tonic THIK-1 current (left) and voltage dependence of respective C100814-inhibited current (right), revealing a reversal potential of ~ − 90 mV close to the predicted K^+^ equilibrium potential (inset, right). **H** Time course of changes in resting membrane potential by C100814 in murine microglia (left) and quantification of effects in comparison to mice lacking THIK-1 (THIK-1 KO) (right). Note the similar degree of depolarization obtained after pharmacological blockade and genetic deletion. **I** Example trace showing the lack of effect of C100814 on baseline current in microglia from THIK-1 KO mice (left) and quantification of effect relative to values before drug application (right). Data information: data indicate mean ± SEM. Dots on bars show number of cells. Data are from four individual patients, and four mice. *P*-values are from unpaired (**C**, **D**, **H**) and paired (**F**, **H**, **I**) Student’s t tests

To examine the electrical membrane properties of microglia in the human brain, we used acutely resected human neocortical tissue of the medial temporal gyrus (Fig. 1A). Whole-cell patch-clamp recordings of microglia in layers 1/2 identified by morphological criteria (see “Methods”) were obtained in acutely prepared brain slices and were compared to microglia in neocortex from adult mice. Perfusion of the cells with a fluorescent indicator dye via the patch solution revealed the typical microglial morphology with highly ramified processes and small somata in the human brain, similar to that seen in mice (Fig. 1A). Human microglia exhibited a high input resistance of several gigaohms and a resting potential of ~ − 40 mV, resembling that of their murine counterparts (Fig. 1B–D). They showed time-independent currents with a nearly linear current–voltage relationship to increasing voltage steps from − 150 to + 60 mV starting from a holding potential of − 60 mV, indicating the absence of voltage-gated ion channels (Fig. 1E). Consistent with their complex morphology, this suggests an apparently nonactivated state of microglia in the human neocortical tissue examined.

In rodents, THIK-1 has been identified as the main microglial K^+^ channel setting the cells’ resting potential [19]. To determine its role in microglia in the human brain, where genetic targeting is not feasible, specific pharmacological tools are indispensable and did not exist until recently [37]. Using high-throughput screening and structural refinement of the identified lead compound, we developed C100814, a small molecule inhibitor that potently blocked THIK-1 (IC_50_ of 0.071 µM) but none of the other tested K^+^ channels in recombinant expression systems (Additional file 2: Fig. S1A–D). When applied at a saturating concentration of 5 µM to acute human brain slices, C100814 rapidly depolarized microglia by ~ 25 mV (Fig. 1F). This was due to suppression of a C100814-sensitive outwardly rectifying membrane current with a reversal potential close to the K^+^ equilibrium potential, consistent with the blockade of tonic THIK-1 K^+^ current (Fig. 1G). The level of depolarization resulting from the application of C100814 was similar to that seen in the genetic deletion of THIK-1 in murine microglia (Fig. 1H). C100814 had no effect on microglial membrane currents in THIK-1 KO mice (Fig. 1I) or other commonly expressed K^+^ channels, such as voltage-gated inwardly rectifying K^+^ channels, which are predominantly present in activated microglia (Additional file 2: Fig. S1E).

Taken together, C100814 is a specific and selective pharmacological tool for inhibiting THIK-1, which in human microglia generates the main tonic K^+^ conductance setting their resting potential.

### Extracellular ATP increases K^+^ efflux from human microglia via THIK-1 and P2X7 receptors

**Fig. 2:**
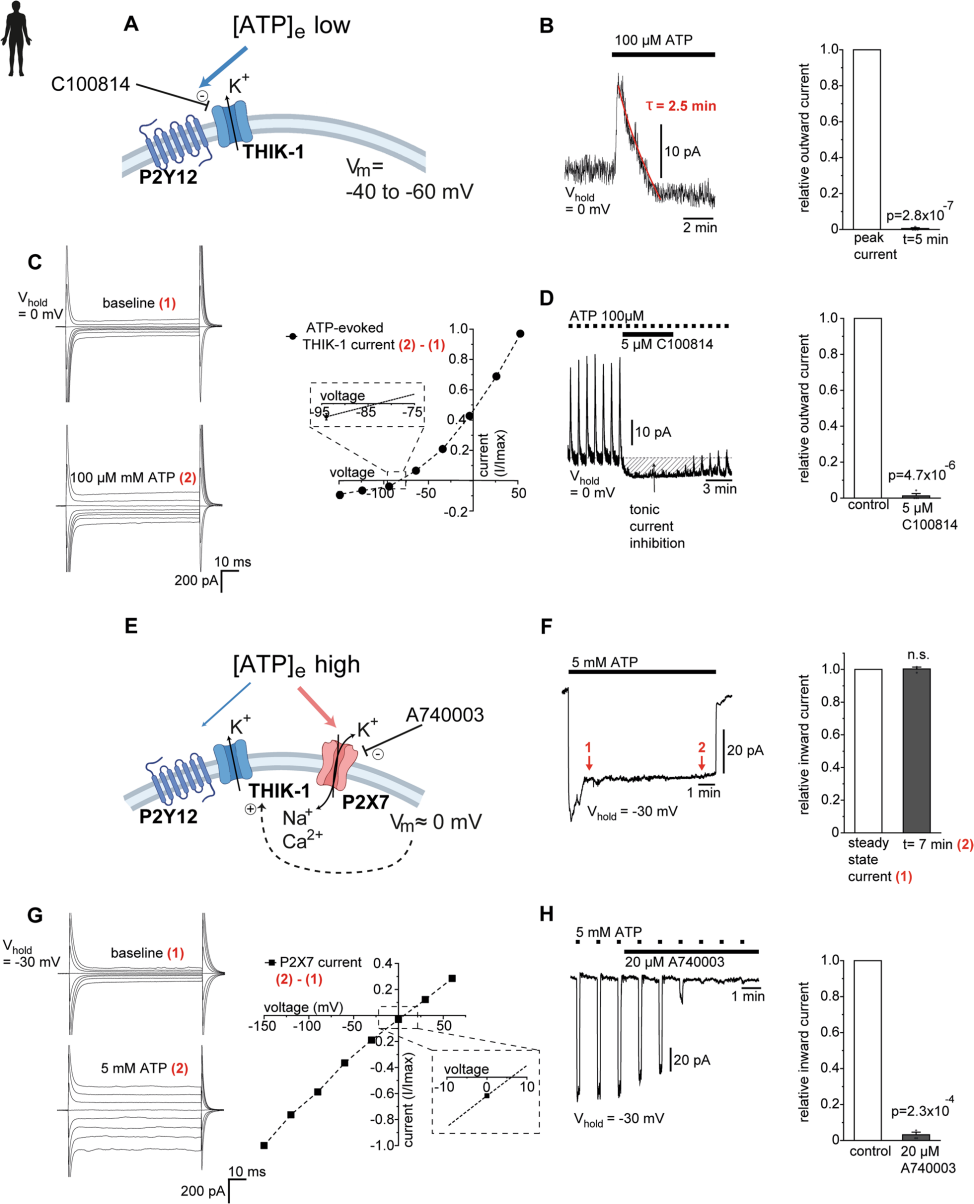
Extracellular ATP increases K^+^ efflux from human microglia via THIK-1 and P2X7 receptors in a concentration-dependent manner. **A** Schematic illustrating the potentiation of THIK-1 K^**+**^ current via metabotropic coupling to high-affinity P2Y12 receptors, which transiently hyperpolarizes microglia to ~ − 60 mV. **B** Specimen outward THIK-1 current elicited by 100 µM ATP (left) showing rapid desensitization in the continued presence of ATP, analysed as residual current present 5 min after peak response (right). Mean current amplitude of ATP-evoked THIK-1 current was 19.25 pA ± 3.7 (*n* = 9). **C** Current profiles of a single microglia to voltage steps from − 150 mV to + 60 mV before and during application of 100 µM ATP (left) and respective voltage dependence of the net ATP-evoked THIK-1 current after subtracting (2) − (1) (right). Inset indicates the reversal potential of ~ − 85 mV, close to the predicted equilibrium potential for K^**+**^. **D** Effect of 5 µM C100814 on repeatedly evoked THIK-1 current by brief pulses of locally applied 100 µM ATP (left). Note suppression of the ATP-evoked and tonic THIK-1 current components (shaded area). Quantification of C100814-induced inhibition of ATP-evoked THIK-1 current, normalised to the mean outward current prior to drug application in the same cell (right). **E** Schematic showing ion channel pathways activated in microglia by exposure to high extracellular ATP concentration (> 1 mM). Additional activation of P2X7 cation channels, depolarizes microglia to ~ 0 mV, which concomitantly enhances K^**+**^ efflux via tonically active THIK-1 channels (dashed arrow) due to an increased driving force for K^**+**^. **F** Time course of microglial membrane current to 5 mM ATP at a holding potential of − 30 mV showing the emergence of a large sustained inward current that does not desensitize in the continued presence of ATP (left). Quantification of the ATP-evoked inward current 7 min after reaching steady state (2), normalised to current at time (1) when steady state was initially reached (right). Mean current amplitude of ATP-evoked P2X7 was − 103.42 pA ± 59.6 (*n* = 5). **G** Current profile of a single microglia to voltage steps from − 150 to + 60 mV before and during application of 5 mM ATP (left) and respective voltage-dependence of the net ATP-evoked P2X7 current after subtracting (2) − (1) (right). Inset indicates the reversal potential of ~ + 5 mV, close to the predicted equilibrium potential of a nonselective cation channel (P2X7). **H** Effect of 20 µM P2X7 antagonist A740003 on repeatedly evoked P2X7 current by brief pulses of locally applied 5 mM ATP at a holding potential of − 30 mV (left). Quantification of A74003-mediated inhibition of ATP-evoked P2X7 current, normalised to the mean inward current before drug application in the same cell (right). Data information: data indicate mean ± SEM. Dots on bars show number of cells. Data are from four individual patients. *P*-values are from paired Student’s t tests

Elevation in extracellular ATP is one of the most potent pathological stimuli and activator of the NLRP3 inflammasome. Because a decrease in intracellular [K^+^] is a key signalling event for this to occur [13], we next focused on ATP-activated ion channels in human microglia that enable the efflux of K^+^ in addition to tonically active THIK-1. Application of ATP induced biphasic changes in membrane voltage in murine microglia depending on the concentration range. Whereas microglia were hyperpolarized to ~ − 60 mV by 100 µM ATP, exposure to 1 mM ATP depolarized their membrane to ~ 0 mV (Additional file 2: Fig. S2A). This implies a concentration-dependent mechanism and involvement of different types of ATP-sensitive ion channels. Hyperpolarizing outward currents, which in mice are generated by metabotropic potentiation of THIK-1 via high-affinity P2Y_12_ receptors [19], were robustly triggered in human microglia by 100 µM ATP (Fig. 2A, B). This current desensitized in the presence of an agonist and had a reversal potential of − 85 mV (Fig. 2B, C), close to the equilibrium potential for K^+^. Consistent with the activation of THIK-1, C100814 dose-dependently blocked the ATP-evoked current along with its tonic current component (Fig. 2D and Additional file 2: Fig. S2B). Thus, similar to rodents, microglia in the human brain are able to sense elevations in ambient ATP, resulting in a transient increase in THIK-1-evoked K^+^ efflux.

A further increase in ATP up to 5 mM produced a large and persistent inward current, which depolarized human and murine microglia to ~ 0 mV (Fig. 2E, F and Additional file 2: Fig. S2C, D). In contrast to the ATP-evoked THIK-1 response, the inward current did not desensitize in the continued presence of an agonist (Fig. 2F) and had an almost linear current–voltage relationship with a reversal potential of 0 mV (Fig. 2G and Additional file 2: Fig. S2E), suggesting involvement of P2X7 receptors. Indeed, application of the P2X7-specific antagonist A740003 [39] abolished this current, with almost no residual current remaining (Fig. 2H and Additional file 2: Fig. S2F). According to transcriptome data, human and rodent microglia may also express P2X4 receptors [40, 41]. However, these were not functionally present in the plasma membrane, as shown by the absence of inward currents upon applying 200 µM ATP and a lack of effect evoked by ivermectin, which potentiates P2X4 receptor-mediated currents (Additional file 2: Fig. S2G) [42]. This finding indicates that the depolarization of microglia by high [ATP] is mediated entirely by P2X7 receptor activation without contributions from other P2X subtypes. Notably, in the presence of P2X7-evoked depolarization to ~ 0 mV, K^+^ efflux via tonically active THIK-1 channels is increased approximately three-fold compared with the resting potential (see Figs. 1G and 2C), suggesting synergistic effects between P2X7 and THIK-1.

These findings show that in microglia in the human brain, elevation of extracellular ATP activates two main K^+^-permeable ion channels depending on agonist concentration: (i) ATP-potentiated THIK-1 K^+^ channels at ≤ 100 µM ATP and (ii) in addition ATP-gated P2X7 receptors that generate a large nondesensitizing cation current at higher ATP concentrations.

### Blockade of THIK-1 suppresses P2X7-mediated IL-1β release from microglia in the human brain

**Fig. 3:**
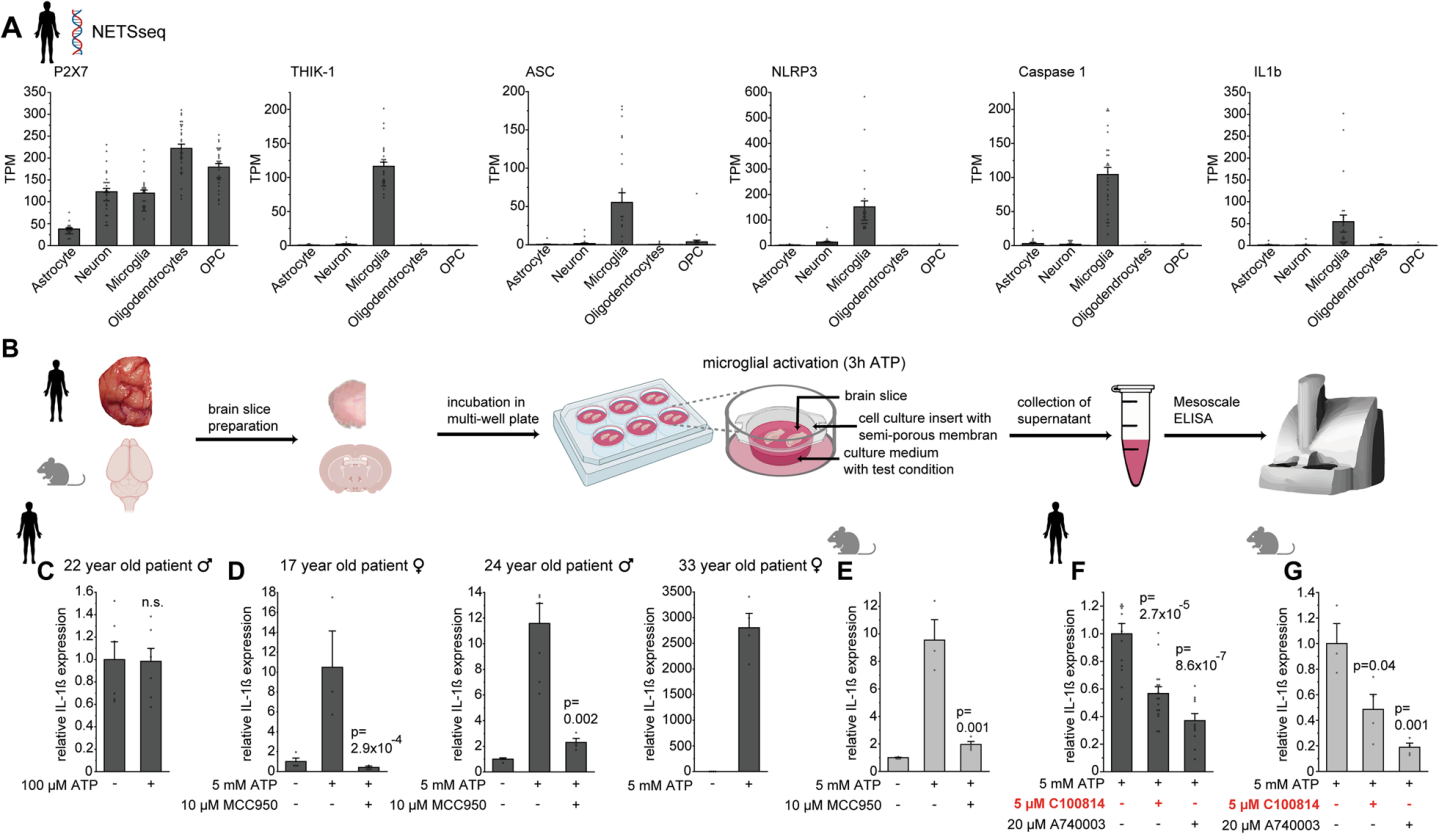
Blockade of THIK-1 suppresses ATP-evoked IL-1β release from microglia in human brain. **A** Comparison of expression levels (mRNA) of P2X7, THIK-1, ASC, NLRP3, Caspase 1 and IL-1β for major CNS cell types analysed by nuclear enriched transcript sort sequencing (NETSseq) of healthy human brain tissue donors (*n* = 32). **B** Workflow illustrating determination of IL-1β levels upon acute activation of microglia in human and murine brain slices by purinergic stimulation (5 mM ATP, 3 h) as a model to simulate acute brain pathology. **C**–**E** Effects on IL-1β release in response to THIK-1 activation by 100 µM ATP (**C**) or in addition by P2X7 activation with 5 mM ATP for 3 individual human donors (**D**), and in comparison to mouse brain slices (**E**). Note the requirement for high ATP (i.e., P2X7 activation) and the dispensability of a priming stimulus to trigger NLRP3-dependent IL-1β release in both human and mouse brain that is abolished by the NLRP3 antagonist MCC950. Data are normalized to control condition and are from four individual patients and four wild-type mice. *P* values refer to 5 mM ATP condition. **F**, **G** Suppression of IL-1β release triggered by 5 mM ATP upon blockade of THIK-1 and P2X7 in human (**F**) and murine brain slices (**G**). Data are normalised to 5 mM ATP condition and are from three individual patients or four wildtype mice. *P*-values refer to 5 mM ATP condition. Data information: data indicate mean ± SEM. Dots on bars show number of slices. *P*-values are from unpaired Student’s t tests

We next investigated the impact of THIK-1 and P2X7 on NLRP3-dependent IL-1β release. Sequencing (NETSseq [36]) analyses revealed that the main determinants required for IL-1β processing including NLRP3, ASC and caspase 1 are almost exclusively expressed in microglia in the human brain (Fig. 3A), indicating microglia as the primary source of IL-1β release. Since our NETSseq data show a high enrichment of microglia-specific markers over monocyte and macrophage markers, these expression profiles essentially apply to microglia rather than peripheral immune cells. Importantly, unlike P2X7, THIK-1 is found almost exclusively in human microglia and is barely expressed in cell types outside the brain (Fig. 3A and Additional file 2: Fig. S3A). Based on the concentration-dependent recruitment of THIK-1 and P2X7 by ATP described above, we tested the effects of 100 µM and 5 mM ATP. This enables purinergic stimulation of THIK-1 alone (100 µM) and in combination with P2X7 (5 mM) (Fig. 3B). Exposure to the lower ATP dose for 3 h triggered no increase in IL-1β levels in human and murine brain slices, regardless of the presence of LPS as a priming stimulus (Fig. 3C and Additional file 2: Fig. S3B). In contrast, application of 5 mM ATP triggered a robust, ≥ tenfold increase in IL-1β concentration in cortical slices from three individual patients as well as mice compared to controls, which was abolished when NLRP3 activation was blocked by 10 µM MCC950 (Fig. 3D, E) [43]. Consistent with the requirement for high [ATP], IL-1β levels were blocked by the P2X7 antagonist A740003 (Fig. 3F, G), with neither A740003 affecting THIK-1 currents nor C100814 affecting P2X7 currents (Additional file 2: Fig. S3C, D). These findings indicate that activation of P2X7, but not THIK-1, is a sufficient determinant to trigger IL-1β release by ATP in human and mouse microglia. Importantly, however, blockade of THIK-1 by C100814 nearly as strongly suppressed ATP/P2X7-evoked IL-1β release, achieving effect sizes between 60 and 90% compared to P2X7 blockade (Fig. 3F, G). Because of the dual role of P2X7 in NLRP3 priming and activation [44–46], P2X7-evoked IL-1β release required no prior priming stimulus, albeit priming with LPS increased total IL-1β level without affecting the relative potency of THIK-1 blockade (Additional file 2: Fig S3E). Inhibition of IL-1β release by C100814 was dose-dependent, both in primary microglia and brain slices, and entirely abolished in THIK-1 KO mice (Additional file 2: Fig. S3F–H). Noteworthy, the widely used THIK-1 inhibitor tetrapentylammonium (TPA) further suppressed the release of IL-1β in THIK-1 KO mice, pointing to its lack of specificity as opposed to C100814 (Additional file 2: Fig. S3I).

Using purinergic stimulation as a robust model to simulate brain pathology, these results demonstrate that C100814-induced blockade of THIK-1 effectively suppresses NLRP3-dependent IL-1β release from microglia in the human brain. Although potentiation of THIK-1 alone (by 100 µM ATP) is not sufficient to induce IL-1β release, THIK-1 activity is necessary for this purpose when a higher, NLRP3-activating concentration of ATP is applied.

### THIK-1, as opposed to P2X7, contributes marginally to K^+^ efflux as a trigger for NLRP3 activation

**Fig. 4:**
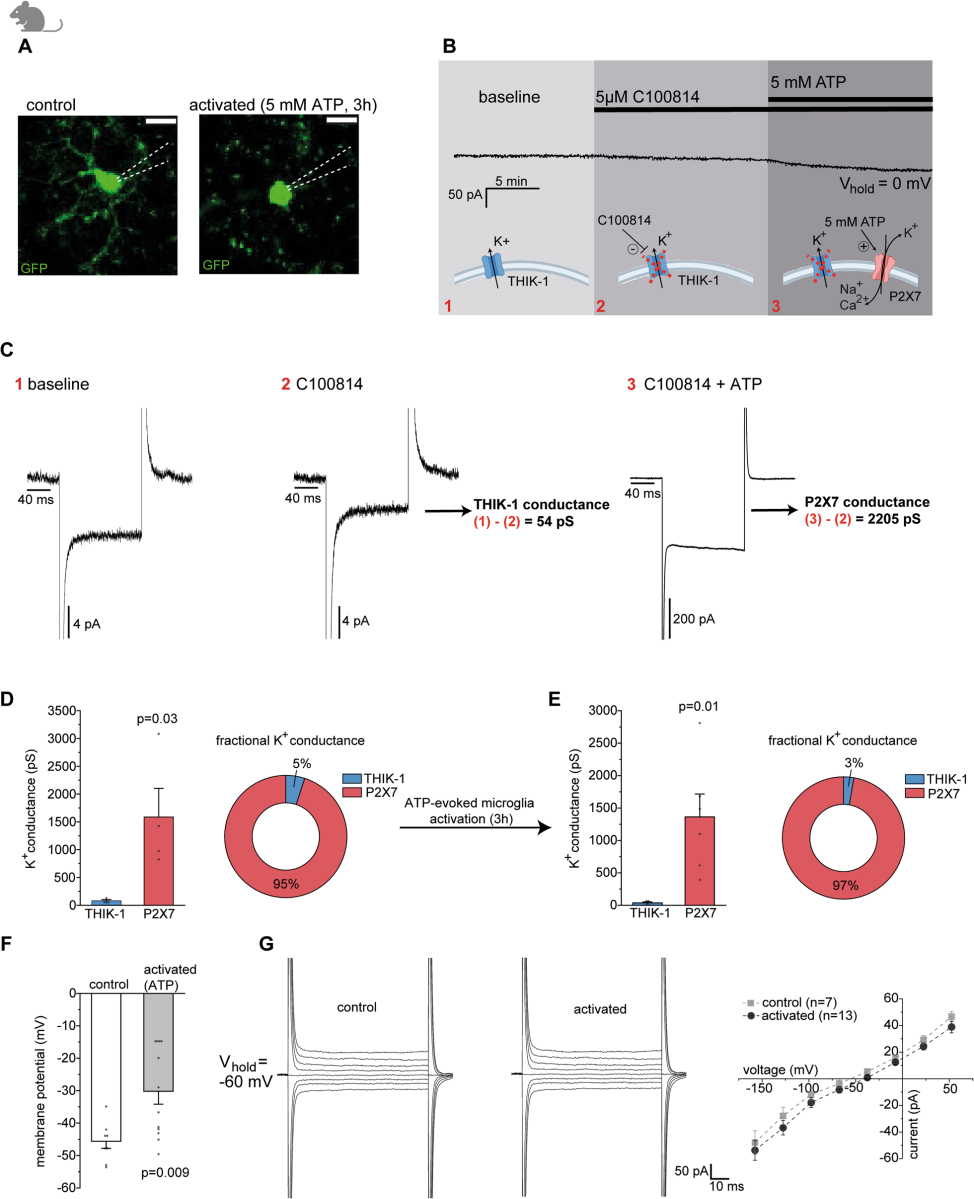
THIK-1, as opposed to P2X7, contributes only marginally to K^+^ efflux for ATP-triggered NLRP3 activation. **A** Confocal images showing GFP-encoded microglia (green) in cortical murine slices before (control) and after microglial activation by ATP (5 mM, 3 h). Scale bar, 10 µm. **B** Exemplary voltage clamp recording of murine microglia at a holding potential of 0 mV (i.e., the approximate reversal potential of P2X7), under similar experimental conditions as those used to study IL-1β release. Schematics depict the stepwise pharmacological isolation of THIK-1 and P2X7 to analyse respective K^+^ conductance ratios from evoked current transients. **C** Current transients in response to 40 mV hyperpolarizing voltage steps of murine microglia (at V_m_ = 0 mV) used to determine THIK-1 conductance by subtracting (1) − (2) and P2X7 conductance by subtracting (3) − (2). Note the different scale on the far right to display the much larger P2X7-evoked current. **D**, **E** Quantification of the individual K^+^ conductance mediated by P2X7 and THIK-1 for nonactivated (**D**) and activated microglia (**E**), considering a K^+^ conductance of 100% for THIK-1 and 45% for P2X7 (see “Methods” for details). Obtained conductance ratios reflect the sustained efflux of K^+^ from microglia in the continued presence of ATP accomplished by nondesensitizing P2X7 and tonic THIK-1 currents (because the ATP-potentiated THIK-1 current is transient and rapidly decays with a tau of 2.5 min). **F** Analysis of the resting membrane potential of nonactivated (control) and activated microglia after purinergic stimulation. **G** Specimen current profiles of a nonactivated (left) and ATP-activated (middle) microglia to voltage steps from − 150 to + 60 mV at a holding potential of − 60 mV. Plot of current–voltage dependencies averaged for all cells per condition (right). Note the more positive reversal potential of activated compared to control microglia and the absence of upregulation of other voltage-gated ion channels after purinergic activation. Data information: data indicate mean ± SEM. Dots on bars show number of cells. Data are from three wildtype mice. *P*-values are from unpaired Student’s t tests

Considering the above findings, it is reasonable to assume that THIK-1 controls NLRP3 activation by contributing to the ATP/P2X7-evoked decrease in intracellular K^+^. Since the latter must drop by at least 50% for NLRP3 activation [13], effective and sustained efflux mechanisms are needed. Because the ATP-potentiated THIK-1 current is transient and quickly returns to baseline, only its tonic current component can contribute to this over time, along with the nondesensitizing P2X7 receptor-mediated current. To analyse the contribution of THIK-1 to the total K^+^ efflux during ATP stimulation, we used electrophysiology, which allows a direct readout of microglial ion conductance at high resolution while maintaining the same experimental conditions used to study IL-1β release. Conductance ratios for THIK-1 and P2X7 were determined in both nonactivated and ATP-activated microglia upon pharmacological isolation (Fig. 4A–C, see “Methods”). This was done using murine microglia, which exhibit almost identical current profiles of both ion channels and contribute to IL-1β release in a similar manner as their human counterparts (see Fig. 2 and Additional file 2: Fig. S2). Cells were voltage-clamped at 0 mV, reflecting the membrane potential that results from ATP-induced P2X7 activation (see Fig. 2G). Surprisingly, this revealed that THIK-1 contributes only 5% to the total ATP-triggered K^+^ efflux, which is almost entirely carried by P2X7 (Fig. 4D). The dominance of P2X7 was even further increased in activated microglia, yielding a contribution of THIK-1 of only 3% (Fig. 4E). Consistently, the decrease in the THIK-1 component in ATP-activated microglia was accompanied by a more depolarized resting membrane potential than that in nonactivated controls (Fig. 4F). This occurred without apparent changes in the current–voltage relationship, ruling out the emergence of other voltage-dependent ion channels (Fig. 4G). Thus, in both unstimulated and activated microglia, THIK-1 K^+^ currents contribute only a small fraction to the total ATP-evoked K^+^ efflux underlying NLRP3 activation, which is carried largely by P2X7 receptors.

These findings imply a previously unknown role of THIK-1 in regulating IL-1β release by a mechanism that is largely independent of K^+^ efflux downstream of P2X7. Based on evidence demonstrating reduced expression of the microglial lysosomal marker CD68 in THIK-1-deficient mice [47] as well as localization of recombinantly expressed THIK-1 and related TWIK2 K+ channels in endosomes and lysosomes [48, 49], an additional intracellular role of THIK-1 in microglia may be considered. Indeed, immunoreactivity of the endosomal marker Rab5 and fluorescence intensity of LysoTracker-labeled lysosomal compartments were markedly reduced in primary microglial cultures from THIK-1 KO mice compared to those from WT mice (Additional file 2: Fig. S4A–D). This may suggest the involvement of THIK-1 in endosomal and lysosomal compartments, which is supported by the intracellular localization of THIK-1 upon recombinant expression and in microglia in the human brain (Additional file 2: Fig. S4E, F).

## Discussion and conclusions

In this study, we investigated the role of THIK-1 and its interaction with P2X7 to limit IL-1β release from microglia in the human brain, a strategy currently pursued to suppress neuroinflammation [37]. Taking advantage of the major pathophysiological impact of microglia in virtually all brain diseases, implementation of this approach requires specific pharmacological agents to block THIK-1, which have not previously existed. By introducing C100814, a newly developed THIK-1-specific inhibitor, we validated its efficacy in suppressing ATP-evoked IL-1β release in human brain tissue using a combination of microglial patch-clamp electrophysiology, immunoassays and nuclear-enriched RNA sequencing. By examining the mechanistic involvement of THIK-1 and P2X7 in ATP-induced IL-1β release, we characterised for the first time the electrical membrane properties of microglia in the human neocortex.

Our study yielded the following main results: (i) C100814 is a potent and selective inhibitor of THIK-1 that controls the negative membrane potential of microglia in the human brain, (ii) an increase in extracellular ATP level only activates THIK-1 and P2X7 as K^+^-permeable ion channels in a concentration-dependent manner, (iii) blockade of THIK-1 strongly suppresses ATP-evoked and NLRP3-dependent IL-1β release via a microglial-specific mechanism due to (iv) selective expression of THIK-1, unlike P2X7, in human microglia, (v) ATP-evoked IL-1β release strictly requires coactivation of P2X7, whereas activation of THIK-1 alone has no effect, and (vi) THIK-1, in contrast to P2X7, contributes only marginally to ATP-evoked K^+^ efflux during NLRP3 activation, implying a mechanistic role of THIK-1 that is largely independent of lowering intracellular [K^+^].

### Impact of THIK-1 on the electrical membrane properties of human microglia

To assess its usability in brain tissue, we first tested C100814 in recombinant systems, demonstrating its potency and selectivity over other K^+^ channels, including Kir2.1 and TWIK-2, which are expressed by microglia. C100814 is therefore superior to the commonly used but nonspecific THIK-1 antagonist TPA and other compounds, which all lack specificity and affect numerous other targets (see Additional file 2: Fig. S3I) [20, 50–52]. This is particularly crucial for studies in acute human preparations where additional genetic targeting is not feasible and mechanistic conclusions rely primarily on pharmacological intervention. To study the effects of C100814-induced THIK-1 inhibition in the human brain, electrophysiological validation in microglia is essential, for which we patch-clamped microglia in human neocortical slices at depths > 60 µm below the slice surface and no later than 5 h after surgical removal of brain tissue to minimise interference from the cutting procedure. This was achieved without fluorescent labeling of microglia via dye-coupled lectins and instead relied on the experimenter being trained to identify microglia based on morphological criteria (which was facilitated by high-resolution differential interference contrast-enhanced infrared video microscopy). All microglia examined had a high membrane resistance and resting membrane potential of − 40 to − 50 mV and showed no voltage- or time-dependent currents, similar to their murine counterparts. Although pathology-mediated influences cannot be excluded, this suggests a nonactivated state of microglia in the neocortical tissue examined. As we found, application of C100814 induced a strong depolarization and blocked ATP-evoked THIK-1 currents elicited by metabotropic P2Y12 receptor coupling [19]. This indicates that THIK-1 is the main K^+^ channel that maintains the negative resting potential of human microglia by mediating tonic K^+^ efflux, which is also susceptible to transient potentiation by extracellular ATP. The resting potential is a crucial electrical parameter of any cell [53] that affects all electrochemically dependent mechanisms. This includes ion gradient-dependent membrane transporters, influx of Ca^2+^, or maintenance of K^+^, Na^+^, and other ionic gradients, including volume regulation, which globally impacts the cells’ functional state. In immune cells, changes in the resting potential can have significant implications for their activation state, as recently shown for peripheral macrophages [54, 55]. Conceivably, membrane depolarization by C100814 may affect the functional and metabolic state of microglia with implications for their immunological profile via a multitude of voltage-dependent mechanisms that are not directly linked to THIK-1-mediated K^+^ flux.

### Therapeutic implications of THIK-1 versus P2X7 inhibition

Apart from P2X7, THIK-1 has recently received particular attention as a potential therapeutic target because of its ability to regulate the release of IL-1β from rodent microglia and macrophages [19, 20]. This is attributed to the proinflammatory role of IL-1β and the implication of the IL-1β-producing NLRP3 inflammasome in numerous neurodegenerative conditions, such as Alzheimer’s and Parkinson’s disease or ALS [56]. A critical involvement of THIK-1 is further suggested by its increased expression in neurodegenerative conditions, as shown in brains from Alzheimer’s and Parkinson’s disease donors [37, 57] and in microglia in ALS models [58, 59]. As a key finding, we have shown that inhibition of THIK-1 by C100814 is capable of potently suppressing NLRP3-dependent IL-1β release triggered by purinergic stimulation. Limiting NLRP3-mediated IL-1β release could be a useful early intervention in neuroinflammatory processes impacting numerous downstream immunological and inflammatory functions, since IL-1β is a master regulator controlling the formation of many other immunomodulatory substances [60, 61]. Through positive auto- and paracrine feedback, IL-1β may further potentiate microglial activation and induce dysfunction of many other brain cells, including neurons, astrocytes, oligodendrocytes and pericytes, overall resulting in a vicious cycle that promotes inflammation [62–66]. Therefore, it can be assumed that antagonizing THIK-1 and P2X7 may have far-reaching effects on the immunological milieu that exceed merely affecting IL-1β levels. In this regard, a significant advantage is that THIK-1, including the main determinants required for NLRP3 signalling, is almost exclusively expressed in microglia in the human brain, as we show, which is in contrast to its more widespread expression in rodents [40, 67]. Similarly, P2X7 is expressed in many cell types other than microglia in the human brain and periphery, suggesting that blockade of P2X7, as opposed to THIK-1, may lead to a broader range of undesirable effects. Moreover, THIK-1 is not expressed in adaptive immune cells and, if at all, only to a very low extent in other innate immune cells (www.proteinatlas.org) [68], thus implying a largely microglial-specific role of THIK-1 in humans considering the entire immune system.

### Mechanistic role of THIK-1 and P2X7 in IL-1β release

Apart from validating THIK-1 as a promising therapeutic target, our study provided important mechanistic insights into NLRP3-dependent IL-1β release from microglia in the human brain. To date, most knowledge of the mechanisms underlying NLRP3 activation and cytokine release is still from studies on peripheral macrophages and other immune cells in vitro, with only scarce information available from human preparations. Upregulation of NLRP3 and IL-1β has recently been shown in acutely resected human hippocampal tissue in response to seizure-like activity [21], although direct stimulation of P2X7 failed to induce IL-1β release from primary human microglia cultures [69]. The latter is contrary to our results and may be due to the culture conditions that are known to significantly alter the genetic profile of microglia [25]. Given that a decrease in intracellular [K^+^] acts as a generally accepted key mechanism for NLRP3 activation by purinergic and other pathological stimuli [13, 14], THIK-1 was hypothesized to regulate NLRP3 activation by promoting K^+^ efflux [19, 20]. However, our results in murine and human brain show that P2X7 is by far the more relevant conduit for K^+^. In addition to THIK-1, P2X7 is the predominant K^+^-permeable ion channel in murine and human microglia gated by ATP. A 60–70% inhibition of P2X7-dependent IL-1β release achieved by THIK-1 blockade was paralleled by it contributing only 3–5% to total K^+^ efflux, making a potassium-based mechanism unlikely, even when assuming nonlinear effects. This also considers the fact that, unlike P2X7, the ATP-evoked THIK-1 current desensitizes rapidly so that only the much smaller tonic THIK-1 potassium current, although augmented at a depolarized membrane voltage, can contribute permanently to K^+^ efflux. Consistently, purinergic activation of THIK-1 alone was insufficient to trigger NLRP3 activation and downstream IL-1β release, which strictly required coactivation of P2X7. In other words, activation of P2X7 reflects the sufficient condition, whereas THIK-1, operating downstream of P2X7, is the necessary condition. This implies that P2X7 functions as a built-in danger switch in microglia, driving NLRP3 signalling only when ATP levels are high enough to reach its activation threshold. ATP-evoked IL-1β release in both mouse and human brain slices did not require a priming stimulus, consistent with findings demonstrating concomitant priming and activation by P2X7 [44–46, 70, 71]. In microglia and macrophages this involves translocation of transcription factor NF-kB to the nucleus and regulation of Toll-Like Receptor signaling [44, 46]. Extracellular ATP is one of the most potent and best characterised NLRP3 activators and can reach ambient levels of ≥ 100 µM in the inflammatory microenvironment [16], although microglia may be locally exposed to much higher concentrations due to their pronounced ATP-dependent chemotaxis [72], giving rise to the formation of specific brain pathology-dependent contacts with ATP release sites at neuronal structures [73]. A similar constellation of a two-pore domain K^+^ channel, TWIK-2, and P2X7 to regulate NLRP3 activation was recently reported in murine bone marrow-derived macrophages, where P2X7 induced enhanced insertion of TWIK-2 into the plasma membrane [74, 75]. In contrast, our results indicate no increase in THIK-1 in ATP-activated microglia in human tissue and instead suggest decreased expression, consistent with a more depolarized resting potential. Emergence of other K^+^ channels, such as inward or delayed rectifying K^+^ channels characteristic of activated microglia, was also not seen, the activation of which would, however, also be greatly abolished at depolarized potentials set by P2X7 activation.

What alternative mechanisms may underlie the effect of THIK-1 on NLRP3-mediated IL-1β release? Apart from the plasma membrane, two-pore domain K^+^ channels are also frequently present intracellularly. Indeed, the other two members of this family expressed in microglia, TWIK-2 and THIK-2, were shown to be localised intracellularly in macrophages and upon recombinant expression [49, 76]. Consistently, both TWIK-2 and THIK-2 do not contribute to the electrical membrane properties of microglia (this study and Madry et al. [19]). Our findings may suggest that THIK-1, apart from being expressed at the plasma membrane, could indeed also play a role intracellularly, as suggested by intracellular THIK-1 immunoreactivity and reduced endo/-lysosomal marker expression in primary microglia from THIK-1 KO mice. This is in agreement with immunohistochemical data from the Human Protein Atlas [68] (www.proteinatlas.org) as well as endosomal localisation of recombinantly expressed THIK-1 [48] and reduced CD68 lysosomal protein expression in THIK-1-deficient microglia [47]. Therefore, blockade of THIK-1 may impair the function of microglial endosomes and lysosomes as critical regulators of NLRP3 function [77], which warrants investigation by future studies. In the context of the mechanisms studied here, this would apply in particular to lysosomes, as P2X7 mediates destabilization of lysosomal membranes and subsequent release of cathepsins into the cytosol [78–81], thereby contributing to the activation of NLRP3 [13, 82, 83].

### Supplementary Information

**Additional file 1.** Methods for additional figures.

**Additional file 2: Supplementary Figure 1.** Chemical structure and pharmacological characterisation of THIK-1 inhibitor C100814. **A** Chemical structure of small molecule THIK-1 inhibitor C100814. **B** C100814 modulation of constitutive activity of hKCNK13 (THIK-1), mKCNK13, hKCNK6 (TWIK2), hKCNK2 (TREK-1), and potassium sulphate-induced hKv2.1 activity upon expression in HEK293 cells examined by a FLIPR thallium flux assay. Data show mean ± SD normalised to controls using the nonselective THIK-1 antagonist tetrapentylammonium (TPA) from 2 independent experiments performed in duplicates. IC_50_ values for human and murine THIK-1 were 0.071 µM and 0.061 µM, respectively. **C** Inhibition of THIK-1-mediated current in hTHIK-1 expressing HEK cells by C100814 using the Qpatch 48 platform, with an IC_50_ value of 0.143 µM. Current amplitudes were determined at the end of each depolarizing voltage step and are shown normalised to TPA controls. Data indicate mean ± SD from 2 independent experiments (yielding 2–3 data points/concentration). **D** C100814 displacement of radioligand binding to THIK-1 using membranes from HEK-hKCNK13 cells. Responses show the average percent inhibition of specific binding of the tool compound (C101505) (± SD) from one experimental run (performed in duplicates). **E** Left: Specimen current profiles of a patch-clamped microglia in human neocortical slices to voltage steps from − 150 to + 60 mV that expresses inwardly rectifying K^**+**^ currents (Kir) in the absence (1) and presence of 5 µM C100814 (2). Right: Current–voltage relationship of the currents before (1) and during (2) C100814 application and after subtracting (2) − (1), showing a lack of effect of C100814. **Supplementary Figure 2.** Extracellular ATP increases K^+^ efflux from murine microglia via THIK-1 and P2X7 receptors similar than their human counterparts. **A** Bidirectional changes in membrane potential of patch-clamped murine microglia held in the voltage follower configuration in response to locally applied 100 µM and 1 mM ATP via a puffing pipette causing hyperpolarization to ~ − 60 mV and depolarization to ~ 0 mV, respectively. **B** Example recording showing the concentration dependence of C100814-mediated blockade of THIK-1 current evoked by repeated local applications of 100 µM ATP (left). Quantification of C100814-sensitive THIK-1 current normalized to the mean ATP-evoked response prior to drug application of the same cell (right). **C**, **D** Single traces showing the change in voltage (C) and current (**D**) in microglia on application of 5 mM ATP. Note the lack of desensitization. **E** Left: Specimen current traces to voltage steps from − 150 to + 60 mV in the presence of 5 mM ATP and blockade of currents upon co-application of 10 µM of the P2X7 blocker A740003. Right: Voltage-dependence of P2X7-mediated currents before and after A74003 application, revealing a reversal potential of ~ 0 mV characteristic of a nonselective cation channel (P2X7). **F** Time course of inhibition upon application of 20 µM A740003 on repeatedly triggered 5 mM ATP-evoked P2X7 current (left), and analysis of P2X7 inhibition normalised to the mean current response before blocker application in the same cell (right). **G** Left: Lack of membrane current in response to 200 µM locally applied ATP in the presence of 20 µM Ivermectin added to the perfusate to facilitate P2X4 receptor activation. Right: Quantification of responses, normalised to the membrane current before agonist application in the same cell. To avoid simultaneously triggered THIK-1 K^**+**^ currents and improve the signal-to-noise ratio, a CsCl-based intracellular patch solution was used for this experiment. Data information: data indicate mean ± SEM. Numbers on bars show tested cells. Data are from 3 (**A**, **B**) and 4 wild type (**C**–**E**) mice. *P*-values are from paired Student’s t tests. **Supplementary Figure 3.** Expression of human THIK-1 and pharmacology of THIK-1-dependent IL-1β release. **A** In addition to NETSseq, RNA expression data from www.humanproteinatlas.com demonstrate specific expression of THIK-1 across different human cell types. **B** Analysis of IL-1β levels upon activation of microglia with 100 µM ATP added to murine slices for 3 h. Prior to purinergic activation, microglia were primed for 3 h by exposure of slices to 1 µg/ml LPS. Data are normalised to the control condition (priming only). **C**, **D** Lack of effect of C100814 on P2X7-evoked current (**C**), and A740003 on ATP-gated THIK-1 current (**D**) in microglia in murine slices, normalised to internal control responses prior to application of respective antagonist. **E** Release of IL-1β upon activation of murine microglia with 5 mM ATP in the presence (black bars) and absence (grey bars) of LPS priming, and blockade by 5 µM C100814 in slices from wild-type mice. Note different scales on the left and right y-axes. **F** Concentration-dependent blockade of IL-1β release by C100814 from LPS-primed primary microglia exposed to K^**+**^-free medium for NLRP3 activation. Data show percent inhibition relative to DMSO-treated microglia (control) and are from 4 independent experiments carried out in duplicates. **G**, **H** IL-1β release upon activation of murine microglia with 1 mM ATP (3 h) and concentration dependent blockade by C100814 and 10 µM MCC950 from LPS-treated slices from wild-type mice (**G**) that is abolished in THIK-1 KO mice (**H**). Data are normalised to the control condition (priming only). **I** Application of 50 µM tetrapentylammonium (TPA) to slices from THIK-1 KO mice further reduces IL-1β levels, indicating unspecific, THIK-1 independent effects of TPA. Data are normalised to the ATP condition. Data information: Data indicate mean ± SEM. Numbers on bars show tested cells (**C**, **D**) or number of slices (**B**, **F**–**H**). Data are from 3 (**C**, **D**) and 4 (**B**, **E**, **G**–**I**) mice. *P*-values are from paired (**C**, **D**) and unpaired (**B**, **E**, **G**–**I**) Student’s t tests. **Supplementary Figure 4.** Apart from the plasma membrane, THIK-1 may also play a role intracellularly. **A** Confocal images showing z-projections of Iba1-labeled primary murine microglia from WT and THIK-1 KO mice (green) co-stained with the endosomal marker Rab5 (red). Scale bar, 25 µm. **B** Analysis of mean Rab5 signal intensity per microglia. **C** Lysotracker-labeled primary microglia from WT and THIK-1 KO mice (red) live imaged by 2-photon microscopy. Scale bar, 50 µm. **D** Analysis of Lysotracker signal intensity per microglia. **E** Confocal image of HEK293 cells recombinantly expressing THIK-1-GFP fusion protein (green). Arrow indicates intracellular localisation of THIK-1 near the apical corner of the cell. Scale bar, 25 µm. **F** Confocal images showing Iba-1-labeled microglia (red) in human neocortical slices and THIK-1 immunoreactivity (green). Arrow points toward the intracellular localisation of THIK-1 predominantly at the apical corner of the cell as shown in orthogonal projections (right). Scale bar, 10 µm. Data information: data indicate mean ± SEM. Numbers on bars show analysed cells. Data from primary microglia were obtained from 4 WT or KO mice. *P*-values are from Mann–Whitney tests.

## Data Availability

Data and materials are available from the corresponding authors on reasonable request.

## Abbreviations

ALS: Amyotrophic lateral sclerosis
NETSseq: Nuclear enriched transcript sort sequencing
NLRP3: NOD-like receptor 3
