# Supplementary material for: Differential contribution of THIK-1 K+ channels and P2X7 receptors to ATP-mediated neuroinflammation by human microglia
Source: J Neuroinflammation. 2024 Feb 26;21:58. doi: 10.1186/s12974-024-03042-6 (PMC10895799; doi:10.1186/s12974-024-03042-6)
Supplement: Supplementary file 1 — Additional file 1. Methods for additional figures. [file 12974_2024_3042_MOESM1_ESM.pdf]

Supplementary material

Synthesis of 5-chloro-*N*-[(3-fluorophenyl)methyl]-2-methoxy-*N*-methyl-pyridine-3-carboxamide (C100814)

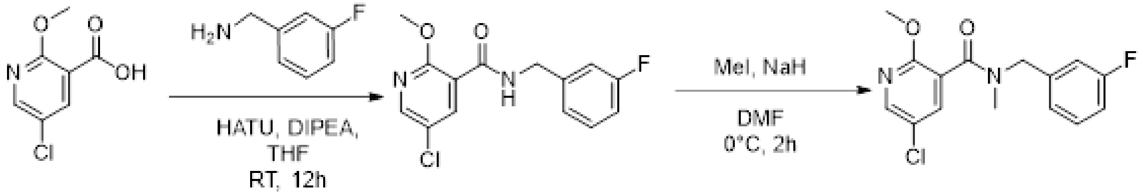

Step 1: A solution of 5-chloro-2-methoxynicotinic acid (0.20 g, 1.07 mmol) in THF (15 mL) was treated with DIPEA (0.309 g, 2.40 mmol), HATU (0.456 g, 1.20 mmol) and (3-fluorophenyl)methanamine (0.15 g, 1.18 mmol) and stirred at RT for 12 h. The mixture was diluted with H_2O (10 mL) and extracted with EtOAc (3 x 20 mL). The combined organic layers were dried over Na_2SO_4 and evaporated under reduced pressure. Purification of the residue by flash chromatography (Davisil silica, 10-20% EtOAc in petrol ether) gave 5-chloro-*N*-(3-fluorobenzyl)-2-methoxynicotinamide (0.15 g, 48%) as an off-white solid.^{1}H NMR (400 MHz, $CDCl_3$): δ 8.52 (d, J = 2.8 Hz, 1H), 8.22 (d, J = 2.4 Hz, 1H), 7.34-7.29 (m, 1H), 7.15-7.11 (m, 1H), 7.06-7.00 (m, 1H), 7.00-6.97 (m, 1H), 4.67 (d, J = 6.0 Hz, 2H), 4.07 (s, 3H). MS m/z: 295.08 $[M + H]^+$.

Step 2: A stirred solution of 5-chloro-N-(3-fluorobenzyl)-2-methoxynicotinamide (0.18 g, 0.60 mmol) in DMF (5 mL) at 0°C was treated with NaH (0.05 g, 1.2 mmol), stirred for 10 min, treated with CH_3I (0.13 g, 0.90 mmol), warmed to RT and stirred for 2 h. The mixture was cooled to 0°C, quenched with H_2O (10 mL) and extracted with EtOAc (3 x 20 mL). The combined organic layers were washed with ice-cold water (10 mL), dried over Na_2SO_4 and evaporated under reduced pressure. Purification the residue by flash chromatography (Davisil silica, 10-30% EtOAc in petrol ether) gave the title compound (0.17 g, 90%) as an off-white solid. ^{1}H NMR (400 MHz, DMSO-d_6 at 90 °C): δ 8.26-8.20 (br s, 1H), 7.85-7.75 (m, 1H), 7.45-7.35 (br s, 1H), 7.25-6.95 (m, 3H), 4.80-4.20 (m, 2H), 3.95-3.80 (m, 3H), 2.95-2.70 (m, 3H); MS m/z: 309.16 (M+H); HRMS calculated for $C_{15}H_{14}ClFN_2O_2$ 308.0728, found; 380.0800. Analy. HPLC: 97.0%.

Primary microglial culture

Microglia were isolated following a modified procedure as described.[1] In brief, newborn wild-type and THIK-1 KO mice aged 2-4 days were euthanized by decapitation. Brain tissue was homogenised and cells were separated by incubation with 0.1% trypsin for 15 min at 37 °C. Cells were then cultivated in poly-L-lysine (PLL) coated flasks at 37°C with 5% CO2 and 95% air in DMEM containing 10% fetal calf serum and 1% penicillin/streptomycin for 7 days in vitro (DIV) until a confluent glial cell layer containing astrocytes and microglia was formed. Microglial proliferation was induced with 5 ng/ml GM-CSF at DIV 7. At DIV 10–14 microglia were harvested by careful shaking of the flasks for 6 min. Cells were cultured at densities of 20.000 to 50.000 per well in PLL-coated 24-96 well plates in serum-free TIC medium containing TGF-β, IL-34 and cholesterol to promote microglial specification.[2] Microglial identity was confirmed by Iba1 immunocytochemistry.

HEK293 and CHO cells

HEK-293 cells were transfected with human THIK-1, TREK-1, TWIK-2, or mouse THIK-1 as previously described[3]. The CHO-Kv2.1 cell line was purchased from Millipore (accession NM_004975) and maintained in F12 (Life Technologies), supplemented with 10% FCSIII (Thermo Scientific) and 500 µg/ml geneticin (Life Technologies). For thallium flux assays, cells were thawed into culture media and seeded into PDL coated 384-well plates (Corning) at 25.000 (human/mouse THIK-1, TREK-1) or 30,000 (TWIK-2) cells/well (25 µl/well). CHO-Kv2.1 cells were seeded into uncoated plates (Corning) at 5.000 cells/well. Plated cells were incubated overnight at 37°C, 5% CO_2 in a humidified atmosphere. For Q-Patch assays, cells were cultured for 7 days prior to assay, dissociated, and diluted to 1-2.5 million/ml in EX-CELL ACF serum free media containing HEPES (25 mM; Life Technologies), penicillin/streptomycin (100 U/ml, 100 µg/ml; Life Technologies) and a trypsin inhibitor (0.04 mg/ml).

IL-1ß release from primary microglia

Cells were primed with LPS (100 ng/ml) for 3.5 h prior to incubation with C100814 or vehicle (0.1% DMSO) for 30 min. K^+ efflux from cells was induced by a complete medium change with an isotonic K^+-free buffer (148 mM NaCl, 10 mM HEPES, 10 mM glucose, 2 mM $CaCl_2$, 1 mM $MgCl_2$ at pH 7.4) in the presence of test compounds or DMSO for 1 h after which supernatants were collected and stored at -20°C until measurement of IL-1β.

Thallium influx assay

A fluorescence-based thallium influx assay (Molecular Devices potassium assay kit) was used to monitor channel activity as previously described[3]. In brief, media was removed from the cell plates and replaced with dye (20 µl/well), the plates were incubated at room temperature in the dark for 60 min. 10 µl from a diluted compound plate (3x FAC in 1.5% DMSO) containing test compound, DMSO vehicle (1.5%, 3x FAC) or tetrapentylammonium chloride (TPA; Sigma, 258962; 1.5 mM in 1.5% DMSO, 3x FAC) was added to the cell plate. This was followed by a 15 min incubation at room temperature in the dark. Using a fluorescent imaging plate reader (FLIPR) tetra plate reader, 15 µl thallium stimulus solution (3 x FAC) was added to the cell plate and the fluorescence signal recorded every second for 1 min (excitation: 470 - 495 nm, emission: 515 - 575 nm). Responses were normalised to baseline and the initial slopes of the thallium induced fluorescence responses (36 - 38 s; baseline corrected) were used to calculate the compound induced inhibition of constitutive (THIK-1, TREK-1, TWIK-2) or potassium sulphate induced activity (Kv2.1).

For the functional wash-off assay, two plates were tested in parallel as described above with an additional assay buffer wash prior to compound addition. After a 15 min compound incubation, plates were either moved to the FLIPR for immediate thallium addition (0 wash) or the plates were washed with assay buffer to remove compound solutions (50 µl/well addition and removal), 30 µl/well assay buffer added and the plates incubated for 60 min, followed by thallium addition on the FLIPR (60 min wash).

Q-Patch automated electrophysiology assay

THIK-1-mediated currents were measured using the Q-Patch 48 automated electrophysiology platform (Sophion, Denmark) as described in Ossola et al.[3]. In brief, experiments were performed at room temperature and in whole cell, single-whole configuration. When whole cell configuration was reached, cells were maintained at a holding potential of -60 mV, followed by a depolarising step to +40 mV for 500 ms to activate THIK-1. Each cell was exposed to 5x liquid additions (minimum of 15 sweeps/liquid addition) with a single compound concentration tested/cell: 1x extracellular (EC) solution (baseline), 2x compound in EC solution, 2x TPA in EC solution. Current amplitude was extracted at the end of the depolarising voltage step and data was rundown corrected using the average of the DMSO negative control cells.

Concentration-response curves were compiled using data from numerous cells within an experiment and compounds were tested in 2 independent experiments, resulting in 2 - 3 data points/concentration.

Competitive binding

A previously disclosed compound C101505, with known THIK-1 inhibition, was tritiated for use in radioligand binding studies (specific activity 14 Ci/mmol). All radioligand binding assays were performed by Gifford Bioscience (Birmingham, UK). THIK-1 cell membrane pellets were prepared, and the assay was run as described in Ossola et al.[3]. In brief, 150 µl of membranes resuspended in assay buffer and 50 µl of C100814 or buffer alone was added to each well of a polypropylene 96 well plate. Following a 30 min, room temperature incubation 50 µl radioligand solution (at approximately the K_D; diluted in assay buffer) was added. The plate was then incubated at 30°C for 120 min with agitation. The incubations were stopped by vacuum filtration onto pre-soaked (0.1% PEI in wash buffer) GF/C filters using a 96-well FilterMate harvester, followed by 6x washes with ice-cold wash buffer. Filters were then dried under a warm air stream, sealed in polyethylene, scintillation cocktail added, and the radioactivity counted in a MicroBeta TriLux counter (Wallac). Non-specific binding was subtracted from total binding for each compound concentration, to give specific binding. The in vitro binding affinity (K_i) of C100814 was calculated from the experimental IC_{50} value using the Cheng-Prusoff equation[4] $K_i = IC_{50}/(1 + ([L]/K_D))$, where [L] was the radioligand concentration in the displacement assay and K_D was the radioligand dissociation constant determined from previous saturation binding studies.

Immunohistochemistry

Slices were fixed overnight in 0.1 M phosphate buffered saline (PBS, 0.9% NaCl) containing 4% paraformaldehyde and 4% sucrose at 4°C. After washing with PBS (3 x 15 min), slices were transferred into blocking solution (10% Normal Goat Serum (NGS) and 0.5% Triton-X in PBS) for 1 h. Slices underwent heat-induced epitope retrieval (HIER) for 20 minutes at 100°C. Endogenous peroxidases were then neutralized with 1% H2O2 for 5 minutes. Slices were incubated with the primary antibodies in PBS containing 5% NGS and 0.3% Triton-X at 4°C for 48-72 hours. Microglia were labeled using monoclonal guinea pig anti-Iba-1 antibody (cat. No. 234 308, 1:1000; Synaptic Systems), and THIK-1 was labeled using polyclonal rabbit anti-THIK-1 antibody (TA338706, 1µg/ml; Origene). Subsequently, slices were rinsed with PBS and then incubated with secondary antibodies in PBS containing 3% NGS and 0.1% Triton-X

overnight, using Alexa Fluor-488 goat anti-guinea pig (1:1000; Jackson, West Grove, USA) for Iba1 and a goat anti-rabbit HRP antibody for THIK-1. For tyramide signal amplification (TSA) of THIK-1 staining, slices were incubated for 10 min with CF®568 Dye tyramide conjugates, mounted using aqueous mounting medium Fluoromount-G (Southern Biotech) and stored at 4°C.

Immunocytochemistry

Coverslips with adherent primary microglia were fixed for 20 minutes in PBS containing 4% paraformaldehyde at room temperature. Cells were blocked and permeabilised in PBS supplemented with 10% NGS and 0.5% Triton-X for 1 h. Cells were then incubated with primary antibody at 4°C overnight. Microglia were labeled using monoclonal guinea pig anti-Iba-1 (cat. No. 234 308, 1:1000; Synaptic Systems). Endosomes and lysosomes were labeled using polyclonal rabbit anti-Rab5 (ab18211, 1:2000; Abcam) and monoclonal rat anti-CD68 (ab53444, 1:500; Abcam). Subsequently, slices were rinsed with PBS and incubated with secondary antibody diluted in PBS containing 3% NGS and 0.1% Triton-X overnight. After washing in PBS, coverslips were mounted using aqueous mounting medium Fluoromount-G (Southern Biotech) and stored at 4°C.

Confocal microscopy

Confocal images were obtained with a 60x objective (oil-immersion, 1.4 NA, 0.13 mm WD) using a Nikon Scanning Confocal microscope (A1Rsi+, Tokyo, Japan) and NIS Elements acquisition software (Nikon). Fluorophores were excited with laser diodes at 488 nm, 561 nm and/or 647 nm. Images were captured sequentially for each fluorophore.

Live imaging of LysoTracker-labeled microglia

Primary microglia were treated with 50 nM LysoTracker ® Red DND-99 for 30 minutes. Lysosomes were imaged using a 25x objective (water, 1.1 NA, 2 mm WD) on an upright Nikon A1R MP+ multiphoton microscope and a Mai Tai Insight DeepSee laser. LysoTracker was excited with the laser tuned to a wavelength of 920 nm and emission was detected using a highly sensitive GaAsP-NDD PMT. For z-stacks, images were captured at 1 µm intervals with a total stack size of 20 µm.

Image analysis of endo- and lysosomes

Raw images were subjected to background subtraction using FiJi software. 3D images were converted into 2D images by maximum intensity projection. Individual region of interests (ROI) for each microglia in the field of view were created based on Iba1 signal as a reference. Using the same imaging parameters for all conditions, mean signal intensities of Rab5 or LysoTracker were determined per individual ROI and subsequently averaged.

Statistical analysis

For thallium influx, radiolabelled binding, and Q-Patch assays, data are presented as mean values ± standard deviation (SD) and were analysed with a four-parameter logistic equation using GraphPad Prism. For thallium and Q-Patch assays, percent inhibition was calculated by normalising data to TPA controls; for the radioligand binding assay, data is expressed as percent inhibition of specific binding of the radiolabeled tool compound.

For assays measuring IL-1β, data are presented as standard error of the mean (SEM) and were analyzed using GraphPad Prism.

Supplementary References

1. Giulian D. Ameboid microglia as effectors of inflammation in the central nervous system. *J Neurosci Res*. 1987;18(1):155-171, 132-133.

2. Bohlen CJ, Bennett FC, Tucker AF, Collins HY, Mulinyawe SB, Barres BA. Diverse Requirements for Microglial Survival, Specification, and Function Revealed by Defined-Medium Cultures. *Neuron*. 2017;94(4):759-773.e8.

3. Ossola B, Rifat A, Rowland A, et al. Characterisation of C101248: A novel selective THIK-1 channel inhibitor for the modulation of microglial NLRP3-inflammasome. *Neuropharmacology*. 2023;224:109330.

4. Yung-Chi C, Prusoff WH. Relationship between the inhibition constant (KI) and the concentration of inhibitor which causes 50 per cent inhibition (I50) of an enzymatic reaction. *Biochemical Pharmacology*. 1973;22(23):3099-3108.
