# Supplementary material for: Differential contribution of THIK-1 K+ channels and P2X7 receptors to ATP-mediated neuroinflammation by human microglia
Source: J Neuroinflammation. 2024 Feb 26;21:58. doi: 10.1186/s12974-024-03042-6 (PMC10895799; doi:10.1186/s12974-024-03042-6)
Supplement: Supplementary file 2 — Additional file 2: Supplementary Figure 1. Chemical structure and pharmacological characterisation of THIK-1 inhibitor C100814. A Chemical structure of small molecule THIK-1 inhibitor C100814. B C100814 modulation of constitutive activity of hKCNK13 (THIK-1), mKCNK13, hKCNK6 (TWIK2), hKCNK2 (TREK-1), and potassium sulphate-induced hKv2.1 activity upon expression in HEK293 cells examined by a FLIPR thallium flux assay. Data show mean ± SD normalised to controls using the nonselective THIK-1 antagonist tetrapentylammonium (TPA) from 2 independent experiments performed in duplicates. IC50 values for human and murine THIK-1 were 0.071 µM and 0.061 µM, respectively. C Inhibition of THIK-1-mediated current in hTHIK-1 expressing HEK cells by C100814 using the Qpatch 48 platform, with an IC50 value of 0.143 µM. Current amplitudes were determined at the end of each depolarizing voltage step and are shown normalised to TPA controls. Data indicate mean ± SD from 2 independent experiments (yielding 2–3 data points/concentration). D C100814 displacement of radioligand binding to THIK-1 using membranes from HEK-hKCNK13 cells. Responses show the average percent inhibition of specific binding of the tool compound (C101505) (± SD) from one experimental run (performed in duplicates). E Left: Specimen current profiles of a patch-clamped microglia in human neocortical slices to voltage steps from − 150 to + 60 mV that expresses inwardly rectifying K+ currents (Kir) in the absence (1) and presence of 5 µM C100814 (2). Right: Current–voltage relationship of the currents before (1) and during (2) C100814 application and after subtracting (2) − (1), showing a lack of effect of C100814. Supplementary Figure 2. Extracellular ATP increases K+ efflux from murine microglia via THIK-1 and P2X7 receptors similar than their human counterparts. A Bidirectional changes in membrane potential of patch-clamped murine microglia held in the voltage follower configuration in response to locally applied 10 [file 12974_2024_3042_MOESM2_ESM.pdf]

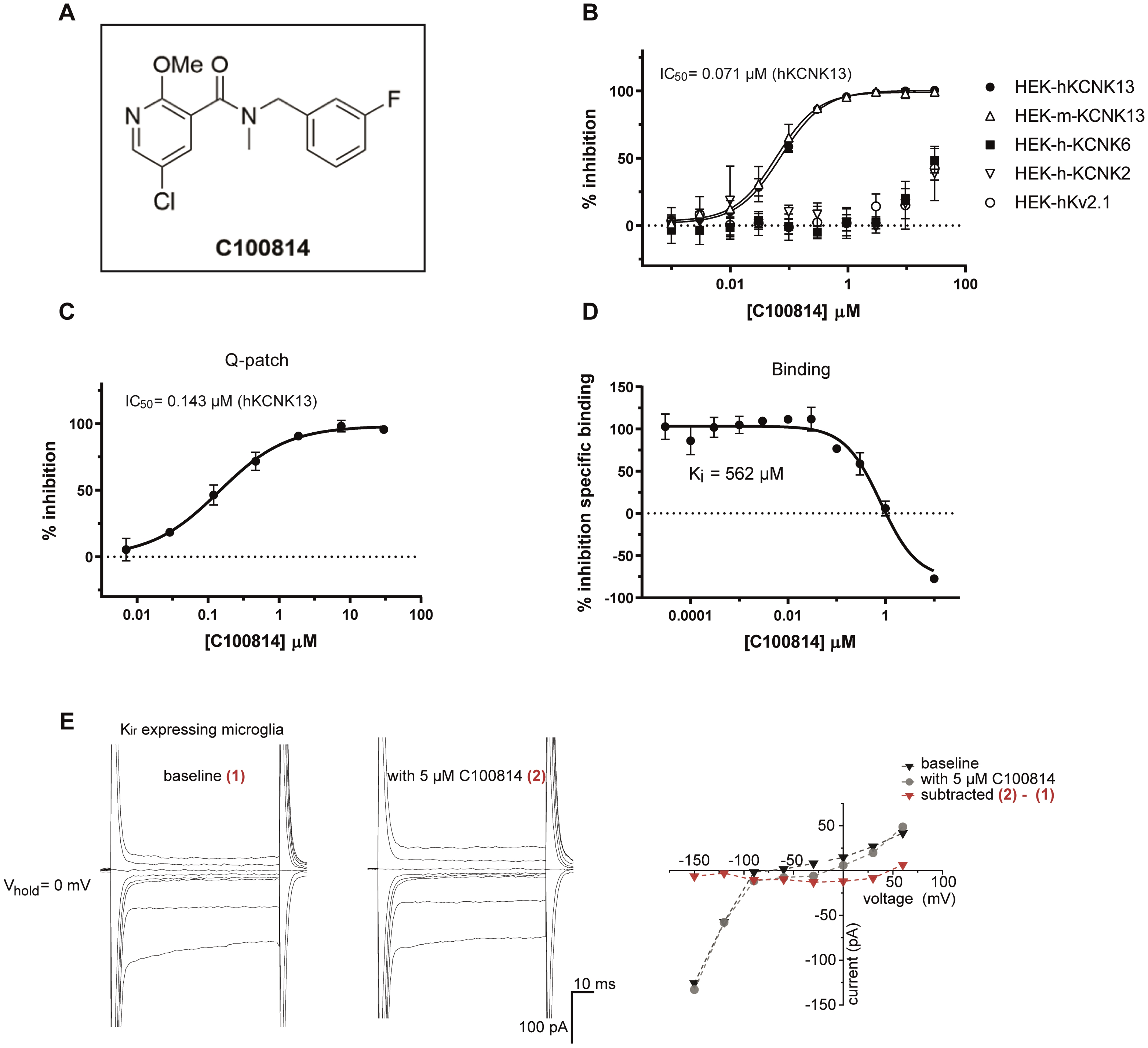

Supplementary Figure 1

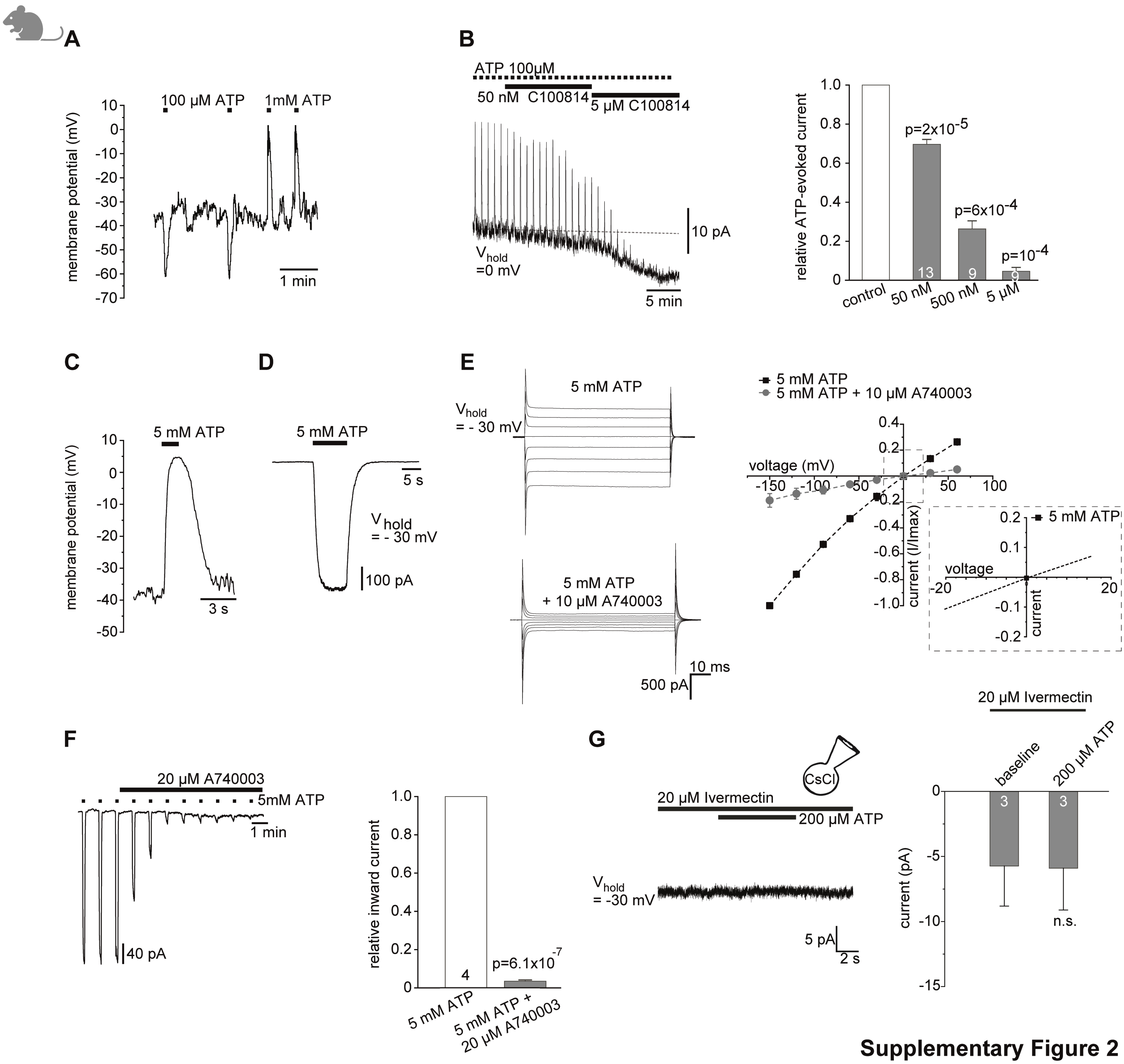

Supplementary Figure 2

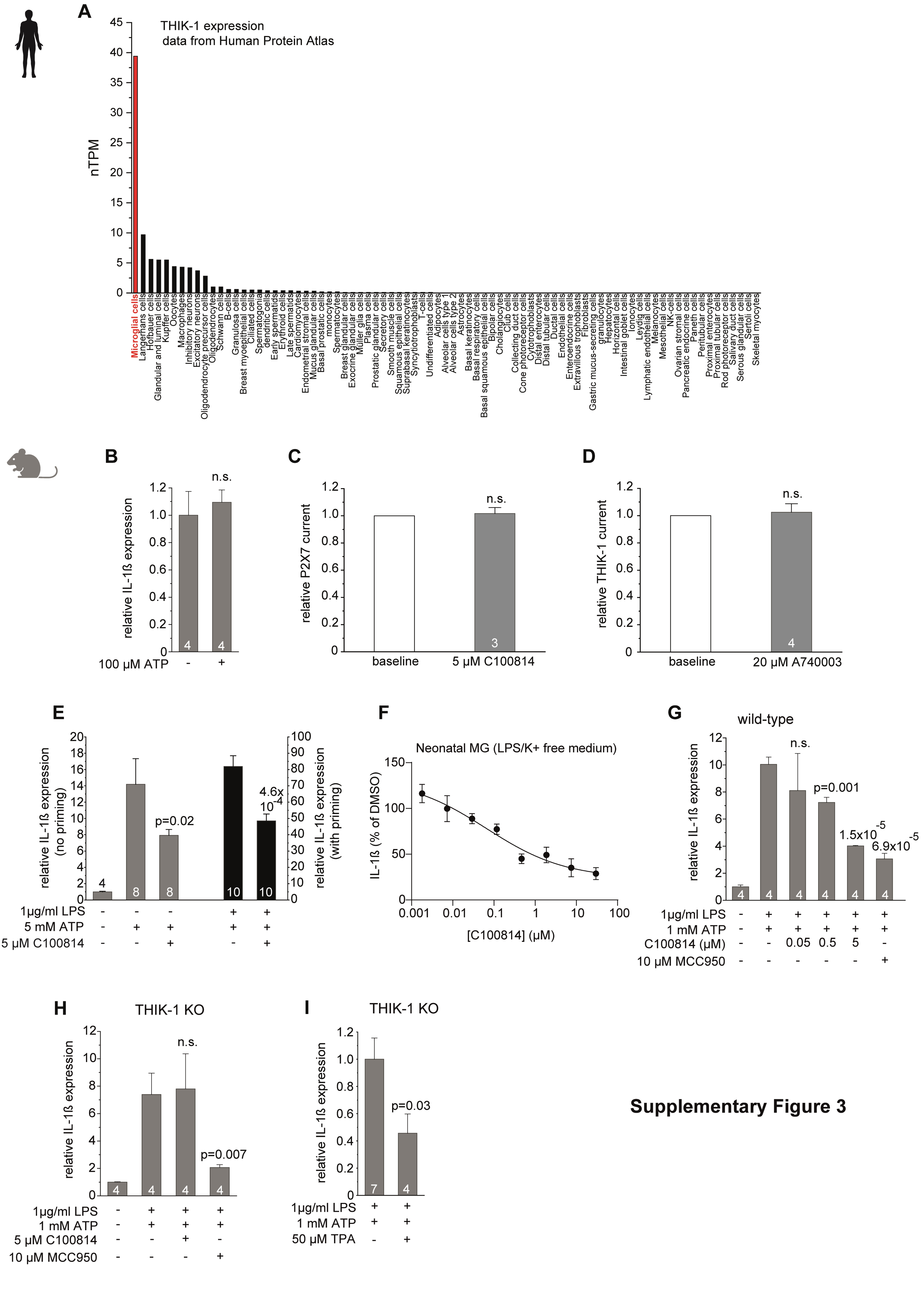

Supplementary Figure 3

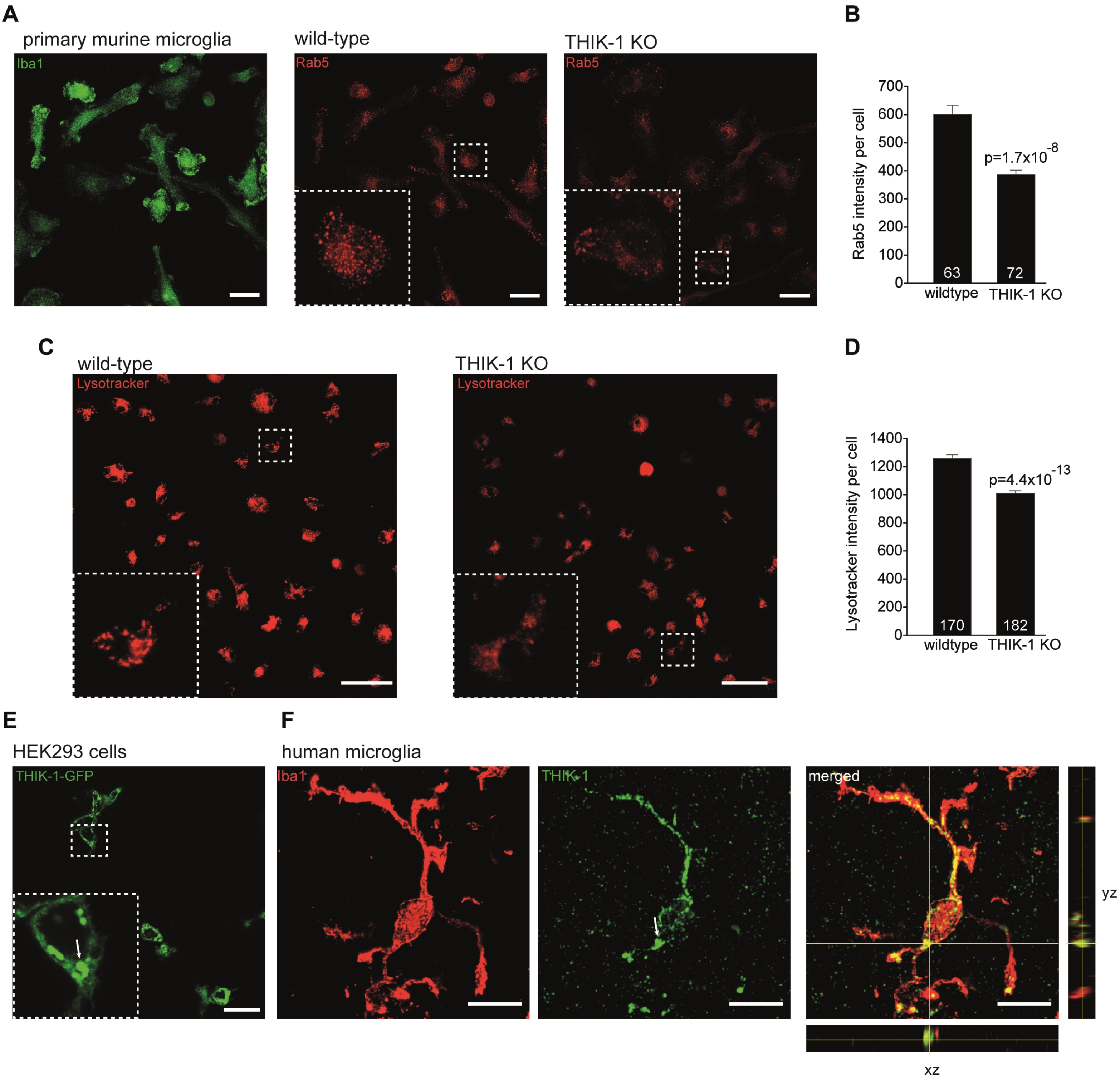

Supplementary Figure 4
